# Heat Stress-Mediated Activation of Immune–Inflammatory Pathways

**DOI:** 10.3390/antibiotics10111285

**Published:** 2021-10-21

**Authors:** Juan M. Cantet, Zhantao Yu, Agustín G. Ríus

**Affiliations:** Department of Animal Science, The University of Tennessee Institute of Agriculture (UTIA), Knoxville, TN 37996, USA; jcantet@utk.edu (J.M.C.); zyu18@vols.utk.edu (Z.Y.)

**Keywords:** heat stress, heat shock protein, inflammation, intestine, nutritional strategies

## Abstract

Physiological changes in animals exposed to elevated ambient temperature are characterized by the redistribution of blood toward the periphery to dissipate heat, with a consequent decline in blood flow and oxygen and nutrient supply to splanchnic tissues. Metabolic adaptations and gut dysfunction lead to oxidative stress, translocation of lumen contents, and release of proinflammatory mediators, activating a systemic inflammatory response. This review discusses the activation and development of the inflammatory response in heat-stressed models.

## 1. Introduction

Homeothermic organisms are sensitive to elevated environmental temperature, leading to hyperthermia, which can compromise the normal functioning of various organs [1]. Ex vivo and in vitro studies conducted to characterize the direct effects of heat have shown that heat directly affects intestinal permeability and function [2,3]. In mammals and birds at the onset of a hyperthermic event, activation of the autonomic nervous system mediated by endogenous catecholamines generates an increase in respiration and heart rate and recirculation of blood with a secondary restricted blood and nutrient flow to the gastrointestinal tract (GIT), consequently altering Ca^+^ and energy cellular metabolism in these tissues and lowering the adaptive capacity to a thermal load [4]. Moreover, a decrease in feed intake to lower the production of metabolic heat and maintain the thermal balance between the heat produced and dissipated is a hallmark of heat stress. Lower nutrient intake triggers adaptations of energy and protein metabolic pathways in addition to those elicited directly by the hyperthermic effect. Heat stress models in pigs [5,6,7], poultry [8,9,10], and ruminants [11,12,13] have been used to characterize changes in the metabolism of macronutrients, but conclusive results have not been reported. Therefore, the phenotype of heat stress includes losses in productivity due to the direct and indirect effects of heat on physiology and metabolism.

Depending on the severity of the heat load, limited nutrient supply and direct thermal effects lead to intestinal dysfunction and can compromise the structure and function of the intestinal mucosa, as shown in studies conducted in rodents and pigs [4,14,15,16,17]. The mechanism by which heat stress alters the intestine is not well understood. However, the pathophysiology of this condition includes loss of tight junction integrity [3,16], translocation of lumen bacteria and their products (i.e., endotoxins [4,16]), and an imbalance between the production of reactive oxygen species (ROS) and their elimination by scavenger systems [4,18]. Consequently, systemic and intestinal inflammation have been observed in livestock and rodents exposed to mild to severe heat [17,19,20,21,22,23] and may be responsible for part of the negative effects of heat stress. The inflammatory response has the potential to impair nutrient absorption, gut health, and the immunological status of the affected organism [18]. In this review, we discuss the cellular and molecular features of the immunophysiological response to heat stress and interventions that aim to prevent harmful effects and the associated inflammatory response.

## 2. How Does Heat Stress Affect the Structure and Function of the Intestinal Mucosa?

### 2.1. Structural Aspects

Exposure to high ambient temperatures may result in structural changes in the small intestine of rodents, poultry, and livestock [4,19,20,23,24,25,26,27,28,29]. The intestinal epithelium is composed of a single layer of cells that lines the inner surface of the small and large intestine, which, in addition to providing a solid protective barrier, functions as a very precise absorptive machinery. In mammals and birds, this cell line folds along its path through the GIT to increase its contact surface and thus its absorptive power, generating villi (raised portions) and invaginations between villi, which are termed crypts. In vivo models for studying gut physiology have shown hyperplastic crypts combined with reduced villus area, suggesting rapid tissue adaptation to nutrient shortages [30,31,32]. Heat stress may affect the intestinal structure by shortening villus height and increasing crypt depth, with a consequent decrease in the villus:crypt ratio, as shown in poultry [19,20,24,25,33], rodents [21,27], and pigs [22,23] (Table 1). The aforementioned effects have been found in different heat-stressed animal models (i.e., ambient temperatures from 33 to 39 °C for 1.5 to 24 h/day, and duration of insult from 1 to 30 days). Furthermore, heat stress has led to epithelial desquamation at the tips of villi and exposure of the lamina propria in the duodenum and jejunum of pigs (i.e., ambient temperature of 40 °C for 2–5 h) [28,29] and in the jejunum and ileum of rats (i.e., ambient temperature of 40 °C for 2 h/day for 10 days [26] or 3 days [34]). Although the direct effect of heat may result in epithelial loss [2], the shift in blood flow away from the gut with a concomitant shortage in nutrient supply to the intestine may contribute to the alterations in intestinal architecture observed in heat-stressed animals.

Changes in the epithelial ultrastructure of the jejunum have been found in animals exposed to heat stress. Electron microscopy analysis revealed that a large amount of inflamed fibrous substances flow out of the hyperthermic rat jejunal epithelium [34]. In pigs and rats, heat stress (40 °C for 2–5 h/day for 10 days) affects epithelial cells of the jejunum, shortens microvillus height and increases the number of mitochondria with shortened internal cristae and secondary lysosomes compared with the jejunum in thermoneutral (TN) animals [26,28,29]. Vacuolization in the epithelium in the jejunum of rats exposed to heat stress has also been reported, possibly in association with the progressive loss of epithelial cells [26]. Although these changes in cellular structure and ultrastructure have been observed in all portions of the SI, it seems that the jejunum has greater susceptibility than other segments of the GIT [28,29,34].

In addition to loss of the epithelium of villi, the metabolic alterations generated by heat stress in the intestinal stem cells at the bottom of crypts may delay epithelial cell turnover and replenishment. Intestinal stem cells exhibit a high regenerative power that ensures the turnover of most mature epithelial cells in less than five days [35]; however, exposure to high temperatures can alter the proliferation and apoptosis of intestinal stem cells. As recently demonstrated by Zhou et al. [36] using in vitro models, continuous heat exposure at 41 °C for 72 h of undifferentiated porcine jejunal epithelial cells inhibits cell proliferation and increases apoptosis via inhibition of the Wnt/β-catenin pathway, the main signal that activates the proliferation of intestinal epithelial cells in the depth of intestinal crypts [37].

### 2.2. Functional Aspects

Stressful events such as exposure to heat can alter the permeability of the intestine to luminal contents (e.g., nutrients and markers) [2,17,38,39]. As alterations in intestinal permeability may reveal changes in absorptive mechanisms (e.g., paracellular pore and leak pathways), this approach is used to assess intestinal function. Gut permeability can be studied by measuring the passage of markers such as fluorescein isothiocyanate-dextran (FITC-D), creatinine, cobalt-EDTA, lactulose and mannitol in vivo and by measuring transepithelial electrical resistance (TER) and permeability to horseradish peroxidase ex vivo and in vitro. Studies have shown that FITC-D increases while TER decreases in the jejunum and/or ileum of pigs and poultry exposed to heat stress [16,19,25,38,40,41,42], indicating an increase in mucosal permeability. In pigs subjected to heat stress (31 ± 1 °C, 12 h/day for 7 days), it was determined that ileum and colon permeability increased, as indicated by a larger lactulose: mannitol ratio [43]. Furthermore, increased intestinal permeability was observed in mice [2,44] exposed to heat stress. In vitro studies have demonstrated increases in permeability or decreases in TER in cultures of epithelial cells, such as intestinal epithelial cell-6 (IEC-6) [44], porcine jejunal cell line (IEC-J2) [43], and human colon-derived crypt-like cells T84 [45] exposed to heat treatments.

Tight junctions (TJs) and adherent junction proteins play a key role in controlling the permeability of the intestinal epithelium [2,46] and in controlling the passage of nutrients via the paracellular space between adjacent cells [47]. Expression of TJ proteins (i.e., occludin and zonula occludens-1 (ZO-1) in the jejunum [19] of chickens and ZO-1 in the jejunum of dairy cows [17]) was reduced during heat stress, and heat stress reduced expression of occludin and claudin-3 in the ileum of pigs [16]. Such an increase in occludin expression was also reported in a study with Caco-2 cells exposed to heat [48]. Gene expression analyses showed higher mRNA expression of claudin-5 and ZO-1 in the jejunum and claudin-1 and -5 and ZO-1 in the ileum in heat-stressed broilers [49]. It was reported that in pigs, heat stress increases the mRNA abundance of occludin, ZO-1 and claudin genes (i.e., pig jejunum after exposure to constant 35 °C for 7 days [23]); in dairy cows, heat stress increases the mRNA abundance of the ZO-1 and claudin-3 genes (i.e., cow jejunum after constant 28 °C for 4 days [17]). However, mRNA levels of occludin, ZO-1, and claudin-1 genes decrease in the jejunum [19,25] of heat-stressed broilers. These contradictory results can be attributed in part to differences between species and studies concerning the duration and severity of heat exposure. It is possible that permeability changes associated with heat stress are mediated by altering expression of TJs in the intestinal epithelium. This relationship has been described in both in vivo and in vitro models of enteritis, aiming to better understand the role of cellular mechanisms to prevent and treat the clinical conditions of humans. Heat stress may lead to changes in TJ expression in the epithelium, but the role of these changes in intestinal permeability remains elusive.

## 3. What Components of the Immune System Are Activated during Heat Stress?

**Figure 1:**
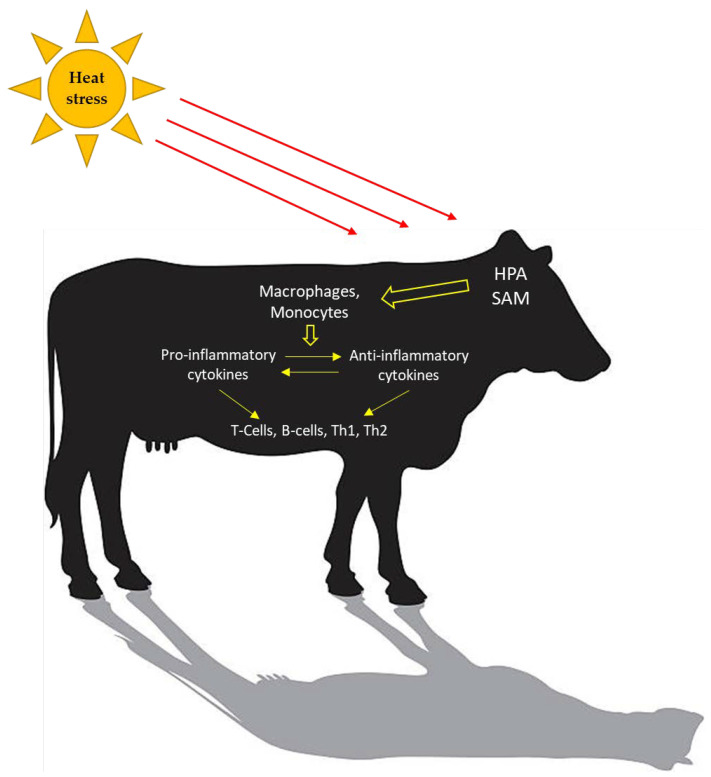
Heat stress activates an immune response. Concentrations of pro-inflammatory and anti-inflammatory cytokines change to maintain homeostasis. The adaptive immune system is cell-mediated (T-lymphocytes; Th1 and Th2) and humoral-mediated (B-lymphocytes).

Innate immunity, and different components of adaptive immunity, can be affected by stress, such as exposure to heat [50]. Although the immunity of higher organisms has a complex framework in which various physical and chemical components participate at molecular, cellular, and tissue levels, innate immunity is the first line of defense against external insults. Adaptive immunity is mediated clonally by T and B lymphocytes and provides tissues with immunological specificity and reaction memory. Heat activates the hypothalamic–pituitary–adrenal (HPA) and the sympathetic–adrenal–medullary (SAM) axes to regulate the response to the stressors and, consequently, elicit changes in the immune response (Figure 1). The release of cortisol during periods of acute stress acts as a stimulus for the immune system; however, during chronic stress cortisol secretion can cause immune suppression. Koch et al. [17] reported a specific effect of chronic heat stress causing infiltration of cells from the adaptive immune system into the lamina propria of the jejunum of lactating dairy cattle.

Studies have shown that the small intestine of broilers [25], pigs [22], and rodents [34] exposed to heat stress impairs innate immunity. In particular, goblet cells are related to mucin production (e.g., mucin-2) and antimicrobial peptides (e.g., protegrin 1–5), chemical components that play a fundamental role in maintaining the integrity of the intestinal mucosa [51]. A decrease in mRNA expression of Mucin-2 and PG 1–5 in the intestinal mucosa of heat-stressed pigs [22] and poultry [25] has been reported. Studies in calves born to cows exposed to heat stress during the last ~60 days of gestation report a low serum concentration and absorptive capacity of immunoglobulin G (IgG) during the first weeks of life [52].

Quinteiro-Filho et al. [53] also found that heat stress decreased the oxidative responses of peritoneal macrophages and the relative weight of the bursa of Fabricius when broilers were exposed to 31 °C (10 h/day for 7 days). Another group of broilers exposed to 36 °C (10 h/day for 7 days) presented a more marked reaction, with reduced thymus and spleen weights in addition to that of the bursa of Fabricius [53]. He et al. [54] found reductions in the growth index of lymphoid organs and an increase in mRNA abundance of several pro-inflammatory cytokines, i.e., interleukin 1 beta, 4, 6 (IL-1β, IL-4, IL-6), and tumor necrosis factor alpha (TNF-α) in the spleen of broilers exposed to 37 ± 2 °C (8 h/day for 14 days).

Sheep exposed to heat stress exhibit increased concentrations of plasma TNF-α, a key mediator of inflammation, and increased total white blood cell, monocyte, and granulocyte counts (40 °C for 12 h followed by 30 °C for 12 h for 30 days) [55]. Expression of proteins associated with the innate immune response, such as haptoglobin, heat shock protein 90-α (HSP90AA1), and the endoplasmic precursor (HSP90B1), is induced in heat-stressed pigs (constant 30 °C for 21 days) [56]. Several reports have shown an increase in the acute-phase protein haptoglobin, serum albumin A, and serum endotoxins in pigs exposed to heat stress for several hours or days [56,57,58]. In agreement with data from pigs, cattle exposed to elevated ambient temperature show greater plasma concentrations of acute-phase proteins (~34 °C 8 h/day for 7 to 21 days) [59,60]. Collectively, these results reveal activation of systemic inflammation during heat stress. Although the role of inflammation is not clear, inflammatory pathway activation may offer opportunities for interventions to treat and prevent the detrimental effects of heat stress [61].

### 3.1. Heat Shock Proteins

**Figure 2:**
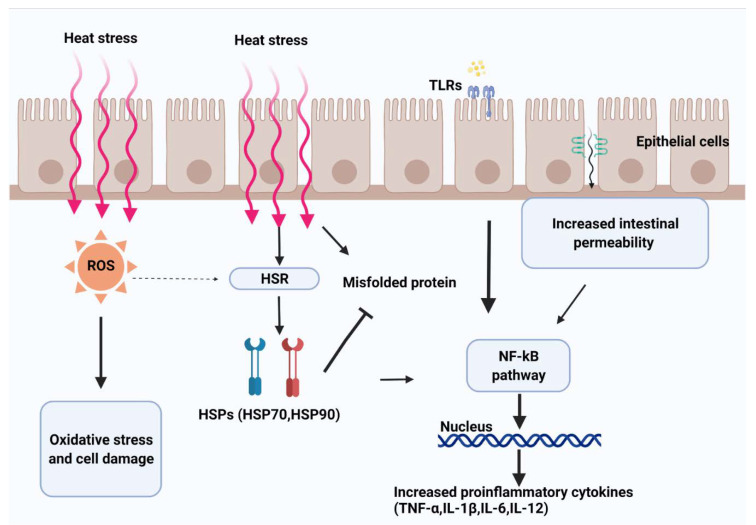
Heat leads to splanchnic hypoxia and metabolic stress. Heat and hypoxia increase the production of oxidants (e.g., reactive oxygen species (ROS)), consequently activating the heat shock response pathway (HSR) to maintain homeostasis. Higher exposure to oxidants increases epithelial permeability by disrupting tight junction proteins. Increased permeability of the intestinal mucosa mediates the passage of luminal contents (e.g., endotoxins and bacteria), activating the immune response mediated by the Toll-like receptor (TLR) signaling pathway and contributing to the host defense system.

Heat shock proteins (HSPs) constitute the heat shock response, acting as signals of cell dysfunction in response to stress stimuli [62,63] (Figure 2). The association between the expression of several HSPs and heat stress accounts for the protective function of these proteins against cell damage [64]. This is because hyperthermia and oxidative stress have negative effects on protein folding, altering their specific biological functions. The heat shock response induces the expression of HSP, which helps prevent or reverse protein misfolding and provides an environment for proper folding (Figure 2). Heat shock proteins are divided into different families according to their molecular weight [65], and of these, HSP90, HSP70, and HSP27 are the most studied in heat-stressed animals [66] (Table 2).

In rodents, HSP70 has protective power in the intestinal mucosa of rats exposed to heat stress. This effect was demonstrated experimentally by inhibiting production of this protein, which, in turn, resulted in augmented mucosal damage of the small intestine [67]. Elevated expression of genes that encode HSP70 and HSP90 was detected in the jejunum of chickens after 15 days of exposure to heat stress [33]. Varasteh et al. [49] also found higher mRNA expression of different HSPs in the intestine of chickens exposed to high ambient temperatures.

Overall, expression of HSPs varies among tissues. For example, the abundance of HSP70 in chicken liver under thermoneutral conditions is double that in muscle; however, under heat stress conditions, HSP70 levels increase at the same proportion in both organs [68]. Similarly, the brains of heat-stressed chickens show almost twice as much HSP70 mRNA than the muscle and liver tissues of the same chicken [68] or rabbit [69]. This tissue specificity characteristic of HSP70 mRNA expression may be associated with the greater sensitivity of essential organs (such as the brain) to heat exposure, especially during severe hyperthermia events [70]. Recent studies in heat-stressed chickens have shown that levels of HSF47, HSF60, and HSP70 increase earlier in the duodenum and jejunum (after 3–6 h of heat exposure) and later in the ileum (after 6–12 h) [71]. Moreover, gene expression of these HSPs after 3 h of acute exposure to elevated temperatures is greater than that of the control in the different portions of the small intestine [71]. Collectively, a better understanding of the role of the heat shock response is of great significance for developing preventive strategies against heat stress.

Heat shock proteins are capable of inducing immune cells, monocytes, macrophages, and dendritic cells to release proinflammatory cytokines such as TNF-α, IL-1β, IL-6, and interleukin 12 (IL-12) [72,73,74,75]. Moreover, studies have reported that HSP activation of immune cells and production of cytokines can be mediated by the NF-κB pathway [72]. Indeed, heat shock proteins may be related to activation of the NF-κB pathway because an increase in expression of NF-κB in the liver [76] and jejunum [33] occurs in birds subjected to hyperthermia (34–37 °C, 8 h/day for 15–32 days). The transcription factor NF-κB regulates multiple aspects of innate and adaptive immune functions: it induces expression of various proinflammatory genes, including those encoding cytokines and chemokines, serving as a key regulator of inflammatory responses [77] (Figure 2). In addition, NF-κB plays a critical role in regulating the survival, activation and differentiation of innate immune cells and inflammatory T cells [78]. Therefore, the role of the heat shock response and NF-κB pathway in the regulation of immune activities is key for characterizing the impact of heat stress on animals.

### 3.2. Toll-Like Receptors

Toll-like receptors (TLRs) are found in animal and plant cells and are typically expressed on the cell surface (TLR1–2, TLR4–6, and TLR10–13) or in endosomes (TLR3 and TLR7–9) of immune (e.g., macrophage, neutrophil, dendritic and natural killer cells) and nonimmune cells [79]. Toll-like receptors have been associated with innate defense against invading microorganisms because TLRs recognize structurally conserved molecules derived from microbes, e.g., bacterial lipopolysaccharides (LPS) [80,81]. In addition, the TLR pathway is activated in response to the binding of endogenous HSP or chromatin-associated protein high-mobility group Box 1 in the intestinal epithelium [82]. Activation of TLR pathways ultimately leads to upregulation or suppression of genes that coordinate the inflammatory response and other events (i.e., cell proliferation and survival and activation of adaptive immunity, Table 2).

Under heat stress conditions, microbial endotoxins may enter the mucosa of the gut and activate TLR pathways, triggering the release of cytokines and coordination of a proinflammatory response (Figure 2). To this end, activation of the TLR pathway has been observed in the GIT of goats [83,84], rodents [34,85], chickens [49], and pigs [86] exposed to heat stress, but the local mechanism and order of events have yet to be determined. Studies in goats exposed to heat stress have shown overexpression of TLR1, TLR3, TLR6, TLR7, TLR8, and TLR10 mRNA in the liver [87]. Furthermore, overexpression of TLR2, TLR4, TLR6, TLR9, and TLR10 mRNA was observed in peripheral blood mononuclear cells (PBMCs) of heat-stressed goats [83]. Although Varasteh et al. [49] found that TLR2 was not affected, mRNA expression of TLR4 was increased in the jejunum and ileum of chickens exposed to temperatures of 38 ± 1 °C for 8 h/day for 5 days. Heat-stressed pigs also exhibit elevated expression of TLR4 in PBMCs on days 1 and 7 at a constant ambient temperature of 35 °C [86]. Nonetheless, heat stress (40 °C 2 h/day for 3 days) reduces expression of TLR2 and TLR4 in the jejunum of rats [34]. In the ruminal epithelium of heat-stressed dairy cows (constant 28 °C for 4 days), activation of TLR4 or the downstream targets of TLR4 (such as IRAK4, p38MAPK, SAPK/JNK, and NF-κB) were not detected, suggesting that heat stress may not affect this segment of the GIT [88]. Collectively, these results suggest an immediate and direct effect of heat and possibly an indirect effect of lumen bacterial antigens on TLR pathway activation in the small intestine; however, conclusive results and the significance of these findings in the pathogenesis of heat-stressed animals have not been clearly defined.

### 3.3. Reactive Oxygen Species

Redox homeostasis can be defined as the capacity of an organism to adapt to and control imbalance between oxidants and antioxidants [89]. Oxidants may be derived from numerous sources, such as mitochondria, xanthine oxidases or other oxidases, and peroxidases. In general, an imbalance in favor of oxidants leads to disruption of redox signaling and control and may eventually lead to molecular and cellular dysfunction or damage. Indeed, imbalance between the production of reactive oxygen species (ROS) and cellular antioxidant defense systems may be one of the main consequences of acute heat exposure leading to oxidative stress [4,90] (Figure 2).

Excessive accumulation of NO, O_2,_ and H_2_O_2_ occurs due to biochemical dysfunction of cellular respiration and metabolism of purines during cell turnover and to nitric oxide synthase activity in response to intestinal hypoxia caused by shifts in blood circulation from splanchnic tissues to the periphery [4]. Wang et al. [91] observed a rapid increase in ROS production in the mitochondria of duodenal, jejunal, and ileal epithelial cells in chickens exposed to 36 °C for 8 h/day. An in vitro study with intestinal epithelial cells demonstrated that exposure to 42 °C for 60 min increases the concentration of ROS and mitochondrial dysfunction and early apoptotic rates [92]. In addition to increased ROS production, heat stress decreases antioxidative enzyme activity in intestinal tissue in rodents [26]. Heat stress may reduce natural antioxidant capabilities, leading to oxidative stress, in the intestines of pigs [40]. In accordance with this, dietary supplementation with selenium and vitamin E alleviate oxidative stress in heat-stressed pigs [40].

Reactive oxygen species act as secondary messengers that mediate the upregulation of the heat shock response pathway during hyperthermia [93]. Reactive oxygen species activation of the heat shock response pathway leads to the breakdown of oxidized intracellular proteins, which appear unfolded or malformed. These signals function to a greater extent with cytoprotection and adaptation to the survival of cells with accumulation of nonnative oxidized proteins after a heat stress event [50].

Heat stress may lead to intestinal damage and translocation of microorganisms and bacterial antigens in pigs, goats, and rats [2,4,15,16,94]. Moreover, the release of ROS plays an important role as part of the innate immunity mediated by neutrophils in response to a stress event such as bacterial translocation. In fact, immune cells release ROS to reduce bacterial colonization and growth and activate the immune response.

In summary, heat stress seems to promote greater production of ROS as a consequence of circulatory system adaptations, but excessive production of ROS via mechanisms related to cell metabolism, division, and immune function may contribute to an imbalance in the oxidative state, leading to oxidative stress and cellular damage [26]. The role of ROS production in livestock exposed to heat stress warrants further investigation to improve our understanding of heat stress-mediated physiology.

## 4. Nutritional Interventions to Avoid or Lessen the Effects of Heat Stress

Dietary interventions have shown a direct beneficial effect on intestinal structure and functionality during heat stress. Some of the dietary compounds with the best prospects for use are detailed below.

### 4.1. Dietary Amino Acids

Dietary amino acids are critical for maintaining the integrity and function of the gut [95]. In recent years, various amino acids, such as L-arginine [22,96] and methionine [97], have been assessed for preventing the negative effects of heat stress on the normal functioning of the GIT. For example, L-arginine acts on numerous metabolic pathways, including protein synthesis and modulation of the immune response [98]. This amino acid can prevent impaired intestinal morphology, limiting villus atrophy in the jejunum and ileum after an inflammatory response [99], and it has been shown to improve the capacity of damaged intestinal hypoxia recovery [100]. The cellular protective activity of dietary L-arginine against a specific heat stress challenge has also been demonstrated [101]. Furthermore, increased intestinal permeability produced by heat stress events [45] are reduced in the presence of dietary L-arginine. This result was shown in heat-stressed mice supplemented with L-arginine, which presented a reduction in excessive intestinal permeability after 4 h of stimulation [102].

The beneficial effects of L-arginine in the development of the epithelial mucosa have been reported in growing piglets supplemented with this amino acid, which presented greater development of villus height throughout the small intestine and crypt depths in the duodenum and jejunum [103]. This response appears not only under physiological conditions but also when the epithelium undergoes a stressful event. With a prolonged thermal stimulus (i.e., 3 days), beneficial effects of L-arginine administration on the integrity of the intestinal structure have been reported, as revealed by a greater villus height with a consequent increase in the villus:crypt ratio in the jejunum of rats. Although the pathways by which L-arginine reduce the impairment generated by hyperthermia in the intestinal mucosa are still uncertain, upregulation of tight junction (ZO-1, occludin, and claudin-6) and adhesion junction (E-cadherin) proteins appears to be key to maintaining intestinal integrity when disrupted by hyperthermia [104] (Table 3). One of the pathways that plays a key role in the anchoring of TJ proteins is activation (through phosphorylation) of AMP-activated protein kinase (AMPK), a key protein involved in energy balance, which promotes expression of ZO-1 and occludin [105,106]. However, phosphorylation of AMPK can be altered by heat stress events [101]. The benefits of L-arginine supplementation in maintaining intestinal integrity after heat stress injury may be channeled by maintaining activation of the AMPK pathway by this amino acid.

Regarding the oxidative effects of heat stress, an increase in radical scavengers such as superoxide dismutase, catalase, and glutathione peroxidase has been observed in the liver and plasma of heat-stressed quails supplemented with methionine [107].

**Table 3:** Effect of nutritional interventions on the structural and functional changes to small intestine during heat stress.

Animal Model	Heat Stress Protocol ^1^	Days of Sampling ^2^	Nutritional Interventions		Intestinal Morphology			Intestinal Barrier Function			Ref. ^14^
			Type ^3^	Product	Tissue	Item ^10^	Change ^11^	Tissue	AJ or TJ Protein ^13^	Change	
Pigs	35 ± 1.0 °C—12 h/day	30	EAA	L-arginine (1% of diet)	Jejunum	VH, V:C	↑	Jejunum	ZO-1 (mRNA)	=	[22]
						CD	=		OCLD (mRNA)	↑	
Rat	40 °C—3 h/day	3	EAA	L-arginine (250 mg/kg BW)	Jejunum	VH	↑	Jejunum	ZO-1, CLDN1 (mRNA)	↑	[101]
						CD	↓				
Rats	40 °C—3 h/day	3	EAA	L-arginine (0.5% of diet)	Jejunum	VH, V:C	↑	Jejunum	ZO-1, OCLD, CLDN6, E-Cadherin (mRNA)	↑	[104]
						CD	=		ZO-1, OCLD, CLDN6, E-Cadherin	↑	
Rats	45 °C—25 min/day	4 h	Prebiotic	Yeast culture ^4^				SI^12^	ZO-1, OCLD, CLDN, JAM-A	↑	[108]
Rats	45 °C—25 min/day	4 h	Prebiotic	Yeast culture		VH, MT	↑				[109]
Broilers	38 ± 1.0 °C—8 h/day	5	Prebiotic	GOS ^5^ (1% of diet)				Jejunum	E-Cadherin	↓	[49]
									CLDN1, CLDN5, ZO-1	=	
								Ileum	E-Cadherin, CLDN1, CLDN5, ZO-1	=	
				GOS (2.5% of diet)				Jejunum	E-cadherin, CLDN5, ZO-1	↓	
									CLDN1	=	
								Ileum	E-Cadherin, CLDN 1, CLDN5, ZO-1	=	
Broilers	33 °C—10 h/day	20	Probiotic	Probiotic A ^6^	Jejunum	VH	↑		OCLD	↑	[19]
						CD, V:C	=		ZO-1	=	
Broilers	35 ± 2 °C—24 h/day	21	Prebiotic	MOS ^7^	Ileum	VH	↓				[110]
						VW, VSA	=				
						CD	↑				
			Probiotic	Probiotic B ^8^		VH	↓				
						VW, CD, VSA	↑				
			Pre + Pro	Combination ^9^		VH	↓				
						VW, VSA	=				
						CD	↑				
Broilers	35 ± 2 °C—24 h/day	42	Prebiotic	MOS		VH, CD, VSA	=				[110]
						VW	↑				
			Probiotic	Probiotic B		VH, VW, CD, VSA	=				
			Pre + Pro	Combination		VH, CD, VSA	↑				
						VW	=				
Rats	40 °C—2 h/day	3	Antioxidant	Ferulic acid (50 mg/kg diet)				Jejunum	E-cadherin, OCLD, ZO-1	↑	[44]

^1^ Heat stress (HS) protocol, including maximum temperature (°C) and intensity (hours of max temperature per day). ^2^ Day when the animals of the experiment (or some of them) were sacrificed and intestine samples were taken to evaluate the structural and/or functional changes of the tissue. ^3^ EEA, essential amino acid. ^4^ Produced by fermentation of *Saccharomyces cerevisiae,* 7 mg/kg BW. ^5^ Galacto-oligosaccharides (Vivinal^©^ GOS syrup, Borculo, The Netherlands). ^6^ Comprised *Bacillus licheniformis*, *Bacillus subtilis*, and *Lactobacillus plantarum* (Chinese Academy of Agricultural Science), 1.5% of diet. ^7^ Mannan-oligosaccharide, 0.5% of diet. ^8^ Comprised *Lactobacillus plantarum, Lactobacillus delbrueckii* ssp. *Bulgaricus, Lactobacillus acidophilus, Lactobacillus rhamnosus, Bifidobacterium bifidum*, and *Streptococcus salivarius* ssp. (Protexin©, Probiotics International Ltd., Somerset, UK.). ^9^ Combination of prebiotic and probiotic described as 7 and 8. ^10^ VH, villus height; CD, crypt depth; V:C, villus height to crypt depth ratio; MT, mucosa thickness; VW, villus width; VSA, villus surface area. ^11^ Change produced by nutritional interventions in an HS environment compared with groups in the same HS conditions but without the nutritional intervention (increase ↑ or decrease ↓ when *p* < 0.05, and without differences, = when *p* > 0.05). ^12^ Small inetstine. ^13^ Adherens junction (AJ) or tight junction (TJ) proteins; ZO-1, zonula Occludens-1; OCLD, occludin; CLDN, claudin; JAM-A, junctional adhesion molecule A. mRNA indicates relative mRNA expression levels of the protein of interest. ^14^ Reference.

### 4.2. Probiotic and Prebiotics

In recent years, probiotics and prebiotics such as yeast extracts or galacto-oligosaccharides (GOSs) have been promoted as feed additives to enhance immunity and GIT health [111] in heat-stressed cows [60,112,113], birds [49], and rats [108]. In most of these studies, the benefits in productive parameters, health, and welfare of the animals as a result of supplementation with pro- and prebiotics were examined.

For example, Liu et al. [114] observed that dietary addition of a yeast-derived β-glucan- and mannan-rich probiotic produced through an insoluble preparation of the cell wall from *Saccharomyces cerevisiae* reduced the expression of HSP70 mRNA in PBMCs from cattle exposed to heat stress. These findings were later supported by results from liver samples collected from heat-stressed cows [115] (Table 4). The authors attributed this downregulation of HSP70 to a decrease in free radical production due to an improvement in antioxidative protection in cows supplemented with the probiotic [114].

Alteration of the intestinal morphology (e.g., decrease in villi height and mucosa thickness) in heat-stressed rats was alleviated by supplementing their diet with a prebiotic fermentative product of *S. cerevisiae* [109]. In addition, the magnitude of the loss of Paneth and goblet cells in the intestinal mucosa was reduced in heat-stressed rats consuming a prebiotic prepared from *S. cerevisiae* [108]. In rats subjected to heat stress, oral supplementation with an *S. cerevisiae* prebiotic protected the integrity of the intestinal barrier, apparently by promoting expression of TJ proteins, i.e., occludin, claudin, ZO-1, and junctional adhesion molecule A (JAM-A) in the intestine [108] (Table 3).

GOS-based prebiotics have been used to maintain intestinal homeostasis, improving the metabolism of the intestinal epithelium in a model of disease in rodents [117,118]. In heat-stressed chickens, oral supplementation with GOSs reversed the increase in mRNA expression of stress biomarkers in the jejunum, such as HSPs, HSFs, E-cadherin, TJ, and TLR4, but it had no effect on reducing elevated mRNA expression of these markers in the ileum of their heat-stressed counterparts [49]. Heat-stressed chickens fed mannan-oligosaccharide probiotic mixtures displayed reduced heat-induced changes in intestinal morphology and intestinal barrier function [19,110]. Protective effects of GOS-based prebiotics in CACO-2 cells exposed to 42 °C for 24 h have also been reported [119]. Treatment with GOSs protected cells against heat stress, as observed by a decrease in heat-induced HSP70 and HSP90 mRNA and protein levels and by suppression of the heat-induced oxidative stress response, which was assessed by mRNA expression of heme oxygenase-1. Furthermore, in this model, heat-induced disruption of the epithelial structure was particularly associated with derangement of E-cadherin, which was mitigated by pretreatment of cells with GOSs [119]. GOSs may have a macromolecule-stabilizing feature that protects cells against oxidative stress and protein carbonylation. Nondigestible oligosaccharides, such as GOSs, stabilize the structure of lipid bilayers and proteins and prevent protein aggregation and oxidative changes in large molecules [119].

### 4.3. Antioxidants

Considering that the oxidative balance is disturbed in heat-stressed animals, dietary supplementation with antioxidants seems to be a logical nutritional intervention. Two common antioxidants, vitamin E and selenium (Se), act synergistically to neutralize free radicals, thus improving preventive antioxidant systems in ruminants [120,121], pigs [40], and poultry [122] exposed to heat stress.

For instance, supplementation of vitamin E and Se can elicit positive physiological responses in heat-stressed animals. Reductions in heat-induced increases in rectal temperature and respiration have been observed in pigs [123] and broilers [124] and in respiration and heart rate in heat-stressed ewes [121]. The improvement in physiological parameters may be related to a reduction in the associated increase in the inflammatory tone and improvement in cellular metabolism [4,121].

Supplementation of vitamin E and Se causes a reduction in oxidative stress (subsequently reducing glutathione peroxidase activity) in the small intestine of pigs exposed to heat stress (35 °C, 8 h/day for 2 days) [40], and supplementation with Se reduces the negative effect of heat stress in growing pigs [116]. In vitro models show the protective effect of Se against exposure to high temperatures at the cellular and molecular levels in porcine small intestinal epithelial cells (i.e., IPCE-J2). Using IPCE-J2 cells, Tang et al. [125] described a heat-induced increase in expression of HSP70, which was reduced due to the presence of Se. In addition, deregulation of the gene and protein expression of claudin-1 and ZO-1 caused by heat stress was reversed in Se treatments [125]. Supplementation with Se reduced expression of proinflammatory cytokines and promoters of oxidative stress, such as IL-6, IL-8, interferon beta (IFN-β), nitric oxide synthase 2 (INOS-2), and monocyte chemoattractant protein-1 (MCP-1) [125].

Furthermore, supplementation with ferulic acid, a powerful antioxidant from the phenolic acid family that is present in numerous vegetables, showed preventive efficacy against thermal injury to the integrity of the intestinal epithelial barrier. This was confirmed in studies with IEC-6 cells, showing an improvement in heat stress-induced TER [44,126]. A decrease in FITC-D associated with the administration of dose-dependent ferulic acid was corroborated in vivo in heat stressed rats [44], reflecting a positive effect on the mucosal membrane integrity of the small intestine by ferulic acid under these conditions. Additionally, decreases in ROS generation were found in IEC-6 cells under heat conditions when increasing the dose of ferulic acids [126]. An improvement associated with ferulic acid with treatment was also found in the ultrastructure of TJ morphology and an increase in TJ proteins (i.e., occludin, ZO-1) and E-cadherin in this cell line [126] and in the jejunum of rats subjected to heat stress [44] (Table 3). Although it is not entirely clear how this compound protects the integrity of the intestinal mucosa, recent evidence supports that the protection against the loss of integrity of the intestinal barrier exerted by ferulic acids in heat stress conditions may be due to activate the PI3K/Akt-mediated Nrf2/HO-1 antioxidant signaling pathway [126].

## 5. Concluding Remarks and Future Perspectives

Physiological changes in animals exposed to elevated ambient temperature are characterized by redistribution of the blood toward the periphery to dissipate heat with a consequent decline in blood flow and oxygen and nutrient supply to the splanchnic tissues. Consequently, metabolic adaptations and gut dysfunction may lead to excessive accumulation of oxidants, translocation of lumen bacteria and endotoxins, and release of proinflammatory mediators. The heat stress phenotype includes activation of a systemic inflammatory response, which may be alleviated by nutritional interventions promoting the maintenance of intestinal homeostasis while reducing systemic inflammation. Future research should aim to elucidate the role of the immune inflammatory response in heat-stressed animals. Nutritional and therapeutic interventions may enhance thermal tolerance to heat by reducing the accumulation of oxidants while maintaining intestinal integrity, but additional data in support of this theory are required.

## Figures and Tables

**Table 1:** Structural changes in the intestinal epithelium of different animals subjected to hyperthermia.

Animal Model	Heat Stress Protocol ^1^	Days of Sampling ^2^	Tissue	Villus Height		Crypt Depth		V:C ^5^		Ref. ^6^
				Change ^3^	% ^4^	Change	%	Change	%	
Broilers	33 °C–10 h/day–20 days	20	Jejunum	↓	18.5	↑	10.0	↓	23.3	[19]
Broilers	39 ± 1 °C–8 h/day–4 days	4	Duodenum	↓	18.4	=	-	↓	50.5	[20]
			Jejunum	↓	17.6	↑	17.0	=	-	
			Ileum	↓	20.2	=	-	↓	40.0	
Broilers	37 ± 2 °C–8 h/day–15 days	15	Jejunum	↓	27.7	↑	28.2	↓	43.1	[33]
			Ileum	↓	24.7	↑	28.8	↓	37.0	
Broilers	37 ± 1 °C —10 h/day–21 days	21	Jejunum	↓	18.6	↑	38.2	↓	39.1	[25]
Broilers	33 ± 0.5 °C–3 h/day–1 day	1	Ileum	=	-	=	-	=	-	[24]
		7 ^†^	Ileum	↓	22.6	↑	14.5	↓	31.4	
Rats	40 °C–2 h/day–10 days	3	Duodenum	↓	21.9	↓	36.4	NA^7^	NA	[26]
			Jejunum	↓	33.1	↓	30.5	NA	NA	
			Ileum	↓	36.1	↓	32.5	NA	NA	
Rats	40 ± 1 °C–1.5 h/day–3 days	3	Jejunum	↓	22.2	=	-	↓	30.6	[27]
Rats	35 ± 1 °C–4 h/day–7 days	7	Duodenum	↓	14.8	=	-	=	-	[21]
			Jejunum	↓	28.9	=	-	=	-	
			Ileum	↓	36.8	=	-	↓	21.0	
Pigs	40 °C–5 h/day–10 days	1	Duodenum	↓	12.3	=	-	=	-	
			Jejunum	↓	20.8	↓	17.4	↓	6.3	[28]
			Ileum	↓	11.2	=	-	=	-	
		3	Duodenum	↓	11.8	↓	23.1	↑	13.3	
			Jejunum	↓	18.8	↓	22.1	=	-	
			Ileum	↓	10.4	=	-	=	-	
Pigs	40 °C–5 h/day–10 days	1	Duodenum	↓	8.8	=	-	NA	NA	[29]
			Jejunum	↓	21.3	↓	15.9	NA	NA	
			Ileum	=	-	=	-	NA	NA	
		3	Duodenum	↓	10.6	=	-	NA	NA	
			Jejunum	↓	22.2	↓	18.7	NA	NA	
			Ileum	↓	9.7	=	-	NA	NA	
Pigs	35 ± 1 °C–24 h/day–7 days	1	Jejunum	↓	14.6	↑	5.2	↓	17.6	[23]
		3	Jejunum	↓	20.4	↑	4.5	↓	23.5	
		7	Jejunum	↓	22.9	↓	4.5	↓	17.6	
Pigs	35 °C–12 h/day–30 days	30	Jejunum	↓	NA	=	-	↓	NA	[22]

^1^ Heat stress protocol, including maximum temperature (°C), intensity (hours of maximum temperature per day) and duration (number of days applying the protocol). ^2^ Day when the animals in the experiment (or some of them) were sacrificed and samples of intestine were taken to evaluate the structural changes in the epithelium. ^†^ In this experiment, heat stress was applied for 24 h and samples of intestine were collected 7 days after the heat stress insult. ^3^ Change elicited by heat stress relative to thermoneutral treatment (increase (↑) or decrease (↓) when *p* < 0.05, and without differences (=) when *p* > 0.05). ^4^ Percentage of change (increase or decrease). ^5^ Villus height to crypt depth ratio. ^6^ Reference. ^7^ Not available.

**Table 2:** Characterization of heat shock proteins (HSP) and Toll-like receptors (TLR) activated during heat stress.

HSP/TLR	Function	Aliases	Localization	Agents/Factors/Domains
HSP90	Protects cells by preventing protein aggregation and enables protein stabilization and trafficking. It also facilitates the activation of numerous regulated proteins.	HSP90AA1, HSP90AB1, HSP90AA2P, HSP90B1	Extracellular, Mitochondrion, Nucleus, Cytosol, Lysosome	HSF1 regulates the activation and release of HSP90 by binding the heat shock elements with the HSP90 promotors.
HSP70	Helps induce protein folding and prevent protein aggregation.	HSPA4, HSPA1A, HSPA8, HSPA14, HSPA1B, HSPA5	Extracellular, Nucleus, Cytosol	Gram-negative bacteria like *E coli* and their proteins.
HSP27	Protect cells from oxidative stress by reducing the ROS through increased production of glutathione.	HSPB1	Endoplasmic reticulum (ER), Cytoplasm	HSP20-like_chaperone, A-crystallin, Alpha-crystallin, ACD, HspB1.
TLR1	Recognizes the pathogen-associated molecular patterns (PAMPs).	CD281 antigen	Plasma membrane, Golgi apparatus	Diacylated and triacylated lipopeptides.
TLR2	Recognizes lipoteichoic acid (LTA).	CD282 antigen	Plasma membrane, Golgi apparatus	PAMPs.
TLR3	Recognizes dsRNA.	CD283 antigen	Plasma membrane, Endosome, Lysosome	Viral dsRNA.
TLR4	Recognizes lipopolysaccharide (LPS).	HToll, CD284	Plasma membrane, Endosome	Triggered by the presence of Ni (2+).
TLR6	Forms heterodimers with TLR2 and recognizes diacyl lipoproteins.	CD286 antigen	Plasma membrane, Golgi apparatus	Cooperates with LY96 and CD14 and acts via MYD88, TIRAP and TRAF6.
TLR7	Recognizes the ssRNA of viruses and synthetic oligoribinucleotides such as imidazoquinoline and imiquimod.	IMD74	Plasma membrane, Endosome, Lysosome	Uridine-containing single strand viral RNAs or guanosine analogs.
TLR8	Recognizes various viral ssRNAs.	CD288 antigen	Plasma membrane, Endosome, Lysosome	GU-rich single-stranded RNA from SARS-CoV-2, SARS-CoV-1 and HIV-1 viruses.
TLR9	Recognizes bacterial CpG-containing oligonucleotides (CpG ODNs).	CD274 molecule	Plasma membrane, ER, Endosome, Lysosome	Unmethylated cytidine-phosphate-guanosine (CpG) dinucleotides.
TLR10	Enables transmembrane signalling receptor ability.	CD290 antigen	Plasma membrane	Acts via the MYD88 and TRAF6 proteins.

**Table 4:** Effect of nutritional interventions on the expression of inflammatory-related genes in different tissues during heat stress.

Animal Model	Heat Stress Protocol ^1^	Days of Sampling ^2^	Nutritional Interventions		Inflammation-Related Genes			Ref. ^14^
			Type ^3^	Product	Tissue	mRNA Relative Expression	Change ^13^	
Rat	40 °C—3 h/day	3	EAA	L-arginine (250 mg/kg BW)	Jejunum	HSF1	↓	[101]
						HSP70, HSP90	↑	
Rats	40 °C—3 h/day	3	EAA	L-arginine (0.5% diet)	Jejunum	NF-κΒ, IL-1β	↓	[104]
Cows	>74 THI—24 h/day	60	Prebiotic	Yeast culture ^4^	PBL ^11^	HSP70	↓	[114]
				Yeast culture ^5^		HSP70	↓	
Cows	>72 THI—24 h/day	28	Prebiotic	Yeast extract ^6^	Liver	HSP27, HSP90	=	[115]
						HSP70	↓	
Broilers	38 ± 1.0 °C—8 h/day	5	Prebiotic	GOS^7^ (1% of diet)	Jejunum	IL-6	=	[49]
						HSP70, IL-8	↓	
						HSF1, HSF3, HSP90	=	
					Ileum	IL-6, IL-8, HSF1, HSF3, HSP70, HSP90	=	
				GOS (2.5% of diet)	Jejunum	IL-6, IL-8, HSF3, HSP70, HSP90	↓	
						HSF1	=	
					Ileum	IL-6, IL-8, HSF1, HSF3, HSP70, HSP90	=	
Pigs	35 °C—8 h/day	2	Antioxidant	Se (0.3 ppm) and Vit E (50 IU/kg)	SI ^12^	HSP70, HIF-1α, IL-8, TNF-α	=	[40]
				Se (0.5 ppm) and Vit E (100 IU/kg)	SI	HSP70, HIF-1α, IL-8, TNF-α	=	
				Se (1.0 ppm) and Vit E (200 IU/kg)	SI	HSP70, HIF-1α, IL-8, TNF-α	=	
Pigs	25–38 °C—24h/day	42	Antioxidant	Se^8^ (0.46 mg/kg diet)	Liver	HSP70, HSP27	↓	[116]
					Kidney	HSP70, HSP27	↓	
					Spleen	HSP70, HSP27	↓	
			Probiotics	Probiotic mixture ^9^ (30 mL/kg diet)	Liver	HSP70, HSP27	↓	
					Kidney	HSP70, HSP27	↓	
					Spleen	HSP70, HSP27	↓	
			Mixture	Se + Probiotic ^10^	Liver	HSP70, HSP27	↓	
					Kidney	HSP70, HSP27	↓	
					Spleen	HSP70, HSP27	↓	

^1^ Heat stress (HS) protocol, including maximum temperature (°C) and intensity (hours of max temperature per day). ^2^ Day when the animals of the experiment (or some of them) were euthanized and samples were collected to evaluate gene expression of inflammation-related genes. ^3^ EEA, essential amino acid. ^4^ Glycerol-enriched yeast culture, produced by fermentation of *Saccharomyces cerevisiae*, contained 76.6 ± 2.21 g/L glycerol and 15.8 ± 0.37 g/L yeast, 31.6 g/day. ^5^ Produced by fermentation of S. cerevisiae grown, contained 33.1 ± 1.03 g/L yeast, 33.1 g/day. ^6^ Extract from yeast cell walls (Zymosan©, Invitrogen), 1:1000 in TMR. ^7^ Galacto-oligosaccharides (Vivinal^©^ GOS syrup, Borculo, The Netherlands). ^8^ Selenium, >90% being organic Se and >75% being selenomethionine. ^9^ Probiotic mixture contained 10^11^/mL CFU of *Lactobacillus acidophilus* and 10^9^/mL CFU of S. cerevisiae. ^10^ Se + Probiotic, a mixture of products 8 and 9. ^11^ Peripheral blood lymphocytes. ^12^ Small intestine, samples of jejunum and ileum. ^13^ Change produced by nutritional interventions in an HS environment compared with groups in the same HS conditions but without nutritional intervention (increase ↑, or decrease ↓ when *p* < 0.05, and without differences, = when *p* > 0.05). ^14^ Reference.
